# Supplementary figures and images for: Identification of key genes and biological pathways in Chinese lung cancer population using bioinformatics analysis
Source: PeerJ. 2022 Jan 31;10:e12731. doi: 10.7717/peerj.12731 (PMC8812315; doi:10.7717/peerj.12731)

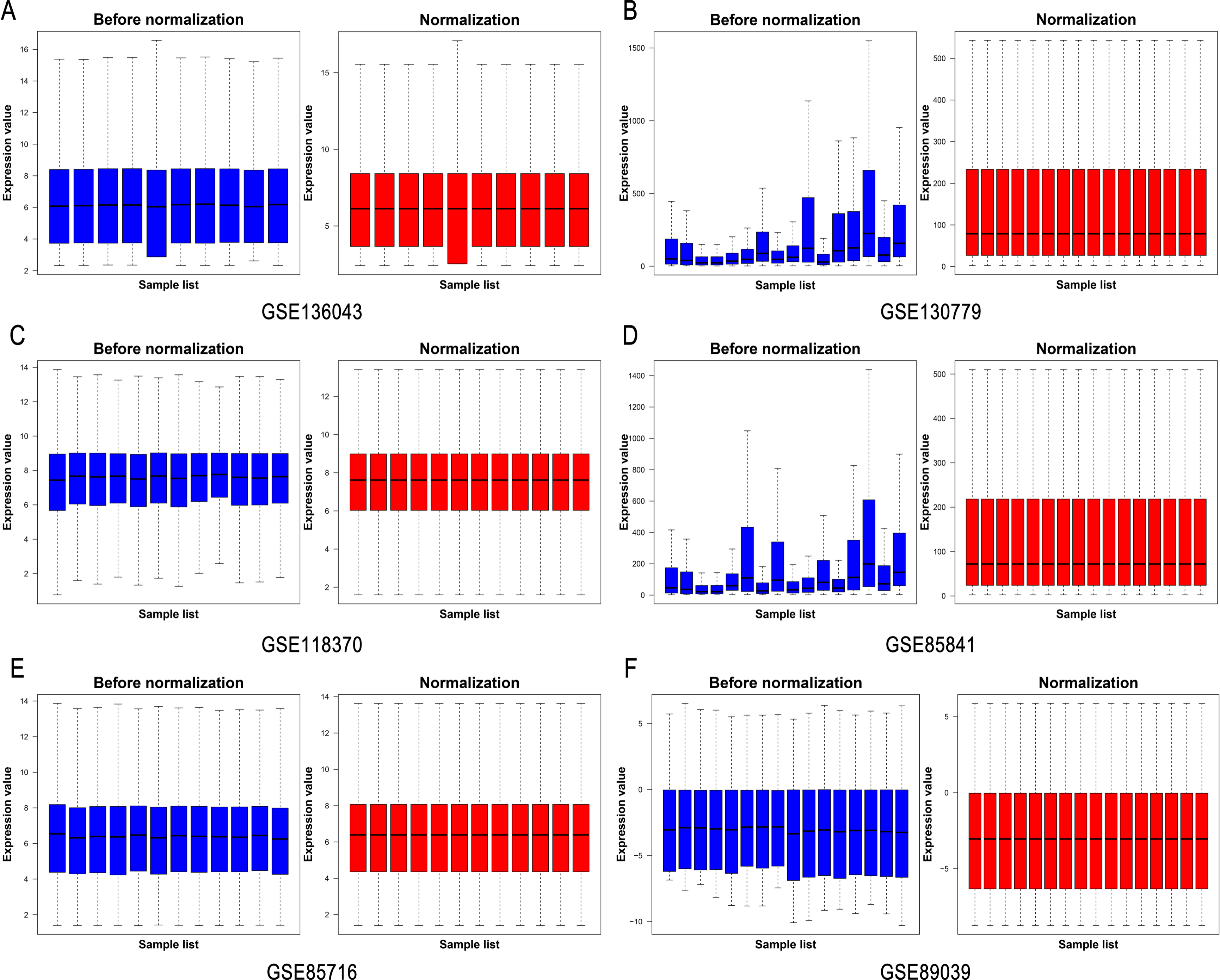

Supplement: Figure S1 — (A) Normalization of the GSE136043 dataset. (B) Normalization of the GSE130779 dataset. (C) Normalization of the GSE118370 dataset. (D) Normalization of the GSE85841 dataset. (E) Normalization of the GSE85716 dataset. (F) Normalization of the GSE89039 dataset. Blue represents data before normalization, and red represents data after normalization. [file peerj-10-12731-s001.pdf]
