# Supplementary material for: Identification of key genes and biological pathways in Chinese lung cancer population using bioinformatics analysis
Source: PeerJ. 2022 Jan 31;10:e12731. doi: 10.7717/peerj.12731 (PMC8812315; doi:10.7717/peerj.12731)
Supplement: Table S1 [file peerj-10-12731-s002.docx]

**Table S1 Clinicopathological characteristics of the 10 patients.**

| **No.** | **Sex** | **Age (years)** | **Pathological type** | **Stage** |
| --- | --- | --- | --- | --- |
| S1 | Female | 77 | Lung adenocarcinoma | T1N0M0 |
| S2 | Female | 69 | Lung adenocarcinoma | T1N0M0 |
| S3 | Male | 57 | Lung adenocarcinoma | T1N0M0 |
| S4 | Female | 67 | Lung adenocarcinoma | T1N0M0 |
| S5 | Male | 70 | Lung adenocarcinoma | T1N1MO |
| S6 | Female | 49 | Lung adenocarcinoma | T1N0M0 |
| S7 | Male | 51 | Lung adenocarcinoma | T1N0M0 |
| S8 | Female | 76 | Lung adenocarcinoma | T1N0M0 |
| S9 | Female | 64 | Lung adenocarcinoma | T1N0M0 |
| S10 | Male | 71 | Lung adenocarcinoma | T1N0M0 |
