# Supplementary material for: Identification of key genes and biological pathways in Chinese lung cancer population using bioinformatics analysis
Source: PeerJ. 2022 Jan 31;10:e12731. doi: 10.7717/peerj.12731 (PMC8812315; doi:10.7717/peerj.12731)
Supplement: Table S2 [file peerj-10-12731-s003.docx]

**Table S2 The primer sequences of 9 hub genes (*CDKN3, MKI67, CEP55, SPAG5, AURKA, TOP2A, UBE2C, CHEK1* and *BIRC5*).**

| **Primers** | **Sequences** |
| --- | --- |
| BIRC5-F | AGCCCTTTCTCAAGGACCAC |
| BIRC5-R | AGTCTGGCTCGTTCTCAGTG |
| CHEK1-F | ATGGGATACCAGCCCCTCAT |
| CHEK1-R | TGGGGTGCCAAGTAACTGAC |
| UBE2C-F | TCTAGCAAGCCCCTTGTGTG |
| UBE2C-R | GGCAGCATGTGTGTTCAAGG |
| TOP2A-F | TTGGAGGCTGTTGAAGCCAA |
| TOP2A-R | CGCGGAGAAGGCAAAACTTC |
| AURKA-F | AATACAGTCCCACCTTCGGC |
| AURKA-R | GGAGCATGTACTGACCACCC |
| CEP55-F | TCTGCTGCAACCTCACGAAT |
| CEP55-R | TCCCGCTGCTGATCATACAC |
| MKI67-F | AGCACGTCGTGTCTCAAGAT |
| MKI67-F | GTTCCCTGAGCAACACTGTCT |
| CDKN3-F | GGACTCCTGACATAGCCAGC |
| CDKN3-R | CTGTATTGCCCCGGATCCTC |
| SPAG5-F | CTGGTAGGGCTTCATGCCAA |
| SPAG5-R | TGCTGGCTCTTGACTGTGAG |
