# Supplementary material for: Identification of key genes and biological pathways in Chinese lung cancer population using bioinformatics analysis
Source: PeerJ. 2022 Jan 31;10:e12731. doi: 10.7717/peerj.12731 (PMC8812315; doi:10.7717/peerj.12731)
Supplement: Table S3 [file peerj-10-12731-s004.docx]

**Table S3 The integrated upregulated genes in lung cancer**

| **Name** | **Pvalue** | **adjPvalue** | **logFC** |
| --- | --- | --- | --- |
| SPP1 | 1.11E-15 | 4.00E-11 | 3.870265291 |
| HABP2 | 2.32E-14 | 8.37E-10 | 2.456063474 |
| CEACAM5 | 3.61E-14 | 1.31E-09 | 3.026189985 |
| COL10A1 | 6.78E-14 | 2.45E-09 | 2.884378477 |
| PITX1 | 2.23E-13 | 8.05E-09 | 2.708043605 |
| CST2 | 3.08E-12 | 1.11E-07 | 2.846833819 |
| SERPIND1 | 3.62E-11 | 1.31E-06 | 2.032072686 |
| TOX3 | 7.94E-11 | 2.87E-06 | 2.592165095 |
| STK32A | 9.20E-11 | 3.32E-06 | 1.988974778 |
| TMEM63C | 9.87E-11 | 3.56E-06 | 2.407282612 |
| CDH3 | 1.07E-10 | 3.88E-06 | 2.124838868 |
| GCNT3 | 3.19E-10 | 1.15E-05 | 1.995050295 |
| CXCL13 | 3.30E-10 | 1.19E-05 | 2.532978075 |
| EEF1A2 | 4.10E-10 | 1.48E-05 | 2.18322078 |
| LRRC31 | 4.65E-10 | 1.68E-05 | 2.235730506 |
| ABCC3 | 5.58E-10 | 2.01E-05 | 2.565762671 |
| TUBB3 | 6.42E-10 | 2.32E-05 | 2.500643059 |
| CXCL14 | 6.80E-10 | 2.46E-05 | 1.936478529 |
| CST1 | 9.34E-10 | 3.37E-05 | 1.92302334 |
| XAGE1A | 1.00E-09 | 3.62E-05 | 2.366485328 |
| RAB26 | 1.02E-09 | 3.69E-05 | 2.140387926 |
| GPT2 | 1.39E-09 | 5.03E-05 | 1.999313885 |
| HS6ST2 | 1.76E-09 | 6.36E-05 | 2.422345109 |
| SCG5 | 1.78E-09 | 6.41E-05 | 1.842849552 |
| CEP55 | 2.27E-09 | 8.20E-05 | 2.156630738 |
| MNX1 | 2.30E-09 | 8.30E-05 | 2.560206718 |
| CDC25C | 2.51E-09 | 9.04E-05 | 1.771620201 |
| GPR110 | 2.54E-09 | 9.16E-05 | 2.073102661 |
| TMPRSS4 | 2.55E-09 | 9.20E-05 | 2.785621922 |
| TOP2A | 2.60E-09 | 9.38E-05 | 2.075213683 |
| ERN2 | 2.61E-09 | 9.41E-05 | 2.499057871 |
| CAPN8 | 3.10E-09 | 0.00011199 | 1.862287123 |
| SPINK1 | 3.16E-09 | 0.000114257 | 2.693452427 |
| LY6D | 3.67E-09 | 0.000132451 | 1.741020886 |
| SBK1 | 4.54E-09 | 0.000164079 | 1.590377648 |
| GJB2 | 4.87E-09 | 0.000175657 | 2.435913363 |
| ETV4 | 4.93E-09 | 0.000178102 | 1.417199941 |
| BIRC5 | 4.99E-09 | 0.000179981 | 1.874404124 |
| HIST1H2AI | 5.07E-09 | 0.000182929 | 1.778319514 |
| ABCA4 | 5.88E-09 | 0.000212336 | 1.842000918 |
| THBS2 | 8.09E-09 | 0.000292134 | 1.722169577 |
| KIF20A | 1.28E-08 | 0.000460692 | 1.825831028 |
| VSTM2L | 1.29E-08 | 0.000465731 | 2.012591493 |
| MEX3A | 1.33E-08 | 0.000478808 | 1.710547277 |
| B4GALNT4 | 1.33E-08 | 0.000479405 | 1.766198573 |
| CENPF | 1.45E-08 | 0.000524631 | 1.616183041 |
| EFNA4 | 1.49E-08 | 0.00053664 | 1.401258017 |
| AGR2 | 1.49E-08 | 0.00053907 | 1.837928194 |
| SULF1 | 1.57E-08 | 0.000566401 | 1.719476018 |
| FAM83A | 1.94E-08 | 0.000701625 | 2.02704542 |
| SLC50A1 | 2.08E-08 | 0.000749372 | 1.449805041 |
| PCP4 | 2.19E-08 | 0.00079198 | 1.805374679 |
| MND1 | 2.33E-08 | 0.000842948 | 1.556759041 |
| KRT6A | 2.36E-08 | 0.000850972 | 1.807716976 |
| HDHD3 | 2.37E-08 | 0.000854292 | 1.320869994 |
| MAP7D2 | 2.40E-08 | 0.000865064 | 1.823931209 |
| METTL7B | 2.59E-08 | 0.000934613 | 1.444661862 |
| IL2RA | 3.24E-08 | 0.001168976 | 1.672923382 |
| B3GNT3 | 3.54E-08 | 0.001279809 | 2.354616793 |
| STIL | 3.58E-08 | 0.001291719 | 1.728576583 |
| SLC22A18 | 3.71E-08 | 0.001341025 | 1.478154762 |
| TRIP13 | 4.10E-08 | 0.001481866 | 1.495268126 |
| MMP12 | 4.48E-08 | 0.001615604 | 2.308823051 |
| PLEK2 | 4.92E-08 | 0.001774978 | 1.642484677 |
| PHLDA2 | 5.30E-08 | 0.001913708 | 1.886279676 |
| IQGAP3 | 5.39E-08 | 0.001944683 | 1.594315257 |
| ACHE | 5.92E-08 | 0.002137532 | 1.928481872 |
| LIMK1 | 6.11E-08 | 0.002207179 | 1.363226215 |
| CDKN2A | 6.61E-08 | 0.002385104 | 1.505742469 |
| UBE2C | 7.29E-08 | 0.002633342 | 1.444652284 |
| IBSP | 7.58E-08 | 0.002737565 | 1.793418446 |
| HOXC9 | 7.84E-08 | 0.002829434 | 1.837190702 |
| ADCK5 | 8.16E-08 | 0.002947721 | 1.489780375 |
| NXPH4 | 8.33E-08 | 0.003008393 | 1.209505025 |
| PLEKHN1 | 8.59E-08 | 0.003101349 | 1.270253078 |
| PPAPDC1A | 9.27E-08 | 0.003347417 | 1.657617663 |
| KIF14 | 9.34E-08 | 0.003372206 | 1.651018662 |
| CENPM | 9.37E-08 | 0.003381254 | 1.68890284 |
| PPAP2C | 9.56E-08 | 0.003451482 | 1.861398278 |
| CDKN3 | 9.78E-08 | 0.003532305 | 1.433696052 |
| GTSE1 | 1.04E-07 | 0.00375585 | 1.772191178 |
| PYCR1 | 1.13E-07 | 0.004064182 | 1.511882818 |
| BARX1 | 1.19E-07 | 0.004285993 | 1.478344458 |
| KIAA1324 | 1.32E-07 | 0.004764958 | 1.374527368 |
| P2RY6 | 1.44E-07 | 0.005215613 | 1.595718824 |
| LOC100130811 | 1.52E-07 | 0.005484338 | 1.660889801 |
| HHIPL2 | 1.54E-07 | 0.005561802 | 2.001412834 |
| SIX1 | 1.70E-07 | 0.006149586 | 1.970961016 |
| NRIP3 | 1.73E-07 | 0.00626145 | 1.4435809 |
| RGS17 | 1.74E-07 | 0.006278595 | 1.7928663 |
| AURKA | 1.78E-07 | 0.006442539 | 1.648153544 |
| RASGEF1A | 1.79E-07 | 0.006451657 | 1.226299143 |
| CTHRC1 | 1.82E-07 | 0.006565178 | 1.528228238 |
| MMP11 | 1.83E-07 | 0.006597579 | 1.983938871 |
| RMI2 | 2.08E-07 | 0.007498301 | 1.386744794 |
| LGR4 | 2.18E-07 | 0.007877133 | 1.353298812 |
| MLXIPL | 2.20E-07 | 0.007945058 | 1.080923319 |
| ADAMTS14 | 2.57E-07 | 0.009272766 | 1.422782187 |
| MELK | 2.65E-07 | 0.009582685 | 1.584379311 |
| SLC44A4 | 2.82E-07 | 0.010174038 | 1.529138519 |
| KCNK5 | 3.00E-07 | 0.010833229 | 1.304463928 |
| HIST2H2AA4 | 3.05E-07 | 0.011018797 | 1.199056277 |
| MKI67 | 3.22E-07 | 0.011611157 | 1.856352999 |
| SPAG5 | 3.31E-07 | 0.01193483 | 1.779957511 |
| MYBPC2 | 3.34E-07 | 0.012051833 | 1.492346426 |
| PAX7 | 3.43E-07 | 0.012390031 | 1.383084343 |
| SLC2A1 | 3.43E-07 | 0.012392133 | 1.153432742 |
| HMGB3 | 3.70E-07 | 0.013354933 | 1.627926235 |
| MMP1 | 3.78E-07 | 0.0136619 | 2.320454401 |
| COL1A1 | 3.81E-07 | 0.013743711 | 1.864345019 |
| AZGP1 | 3.85E-07 | 0.013914323 | 1.909110464 |
| XDH | 3.89E-07 | 0.014035603 | 2.083987503 |
| SLCO1B3 | 3.89E-07 | 0.014044712 | 1.198777322 |
| ARNTL2 | 4.13E-07 | 0.014896036 | 1.483729104 |
| CP | 4.23E-07 | 0.015265723 | 2.26633972 |
| ANO9 | 4.52E-07 | 0.01632896 | 1.488448627 |
| ENPP3 | 4.89E-07 | 0.017646965 | 1.284422412 |
| LSR | 5.39E-07 | 0.019457029 | 1.178242089 |
| ADAMTS16 | 5.51E-07 | 0.019895983 | 1.569514242 |
| ITPKA | 5.54E-07 | 0.019991865 | 1.325225333 |
| DNAJC12 | 5.71E-07 | 0.020600271 | 1.426014516 |
| INCENP | 5.73E-07 | 0.020703093 | 1.150584941 |
| B4GALNT3 | 5.90E-07 | 0.021292303 | 1.216084357 |
| ANKRD22 | 6.19E-07 | 0.022352725 | 1.733050673 |
| DNAH14 | 6.60E-07 | 0.023828268 | 1.440870763 |
| SLCO5A1 | 6.81E-07 | 0.02459158 | 1.337262806 |
| HIST1H3B | 6.84E-07 | 0.024686779 | 1.452299929 |
| TDRKH | 6.89E-07 | 0.024876784 | 1.244530464 |
| C6orf141 | 6.98E-07 | 0.025182476 | 1.710737619 |
| SMPDL3B | 7.11E-07 | 0.02565772 | 1.216699388 |
| PDIA4 | 7.21E-07 | 0.026018729 | 1.500629962 |
| SLC7A5 | 7.23E-07 | 0.026086616 | 1.662858067 |
| WFDC2 | 7.45E-07 | 0.026895759 | 1.546416167 |
| SYCE3 | 7.56E-07 | 0.027280984 | 1.180406098 |
| SPAG4 | 7.74E-07 | 0.027926797 | 1.513250829 |
| SEZ6L2 | 7.74E-07 | 0.027926797 | 1.464675144 |
| SERINC2 | 7.84E-07 | 0.028321468 | 1.346345623 |
| BMP8A | 8.59E-07 | 0.030998178 | 1.198506001 |
| CATSPERB | 8.65E-07 | 0.031210517 | 1.362341901 |
| AIM2 | 8.80E-07 | 0.031786536 | 1.525881702 |
| RAD54L | 8.89E-07 | 0.032077708 | 1.380007619 |
| GALNT14 | 8.95E-07 | 0.032302673 | 1.234720659 |
| CCNO | 9.03E-07 | 0.032598686 | 2.045874926 |
| LEPREL4 | 9.11E-07 | 0.032896956 | 1.228340888 |
| TMEM92 | 9.66E-07 | 0.034891685 | 1.123322869 |
| STAP2 | 9.95E-07 | 0.035926226 | 1.003786809 |
| PVRL1 | 1.03E-06 | 0.037362188 | 1.238713466 |
| SPDEF | 1.03E-06 | 0.037362188 | 1.421547227 |
| LOC100131043 | 1.04E-06 | 0.037651356 | 1.214234397 |
| MMP13 | 1.07E-06 | 0.038660827 | 1.908539683 |
| TMPRSS11E | 1.14E-06 | 0.041136117 | 1.289252163 |
| PADI1 | 1.16E-06 | 0.041738594 | 1.410460338 |
| GJB1 | 1.25E-06 | 0.045208132 | 1.042477019 |
| TMEM61 | 1.26E-06 | 0.045650563 | 1.202916694 |
| MMP9 | 1.27E-06 | 0.045787444 | 1.297297471 |
| LEMD1 | 1.28E-06 | 0.046243847 | 1.579466088 |
| CAPN12 | 1.30E-06 | 0.046766113 | 1.651636312 |
| SALL4 | 1.31E-06 | 0.04724693 | 1.634049915 |
| CHEK1 | 1.33E-06 | 0.048062391 | 1.26241868 |
| B3GNT6 | 1.36E-06 | 0.049066181 | 1.501703014 |
