# Supplementary material for: Identification of key genes and biological pathways in Chinese lung cancer population using bioinformatics analysis
Source: PeerJ. 2022 Jan 31;10:e12731. doi: 10.7717/peerj.12731 (PMC8812315; doi:10.7717/peerj.12731)
Supplement: Table S4 [file peerj-10-12731-s005.docx]

**Table S4 The integrated downregulated genes in lung cancer**

| **Name** | **Pvalue** | **adjPvalue** | **logFC** |
| --- | --- | --- | --- |
| CLDN18 | 2.32E-19 | 8.39E-15 | -4.131675112 |
| AGER | 1.19E-14 | 4.30E-10 | -3.421784457 |
| RTKN2 | 2.13E-14 | 7.69E-10 | -3.295132038 |
| ADAMTS8 | 2.67E-14 | 9.62E-10 | -3.762417126 |
| FIGF | 3.08E-14 | 1.11E-09 | -3.383195712 |
| GRIA1 | 5.47E-14 | 1.97E-09 | -3.336061048 |
| TMEM100 | 9.22E-14 | 3.33E-09 | -3.408355884 |
| SGCG | 1.32E-13 | 4.75E-09 | -3.929608376 |
| LYVE1 | 1.54E-13 | 5.55E-09 | -2.862222151 |
| STXBP6 | 2.01E-13 | 7.26E-09 | -2.818676015 |
| FAM107A | 2.60E-13 | 9.40E-09 | -3.445549783 |
| ADRB1 | 3.61E-13 | 1.30E-08 | -3.028475083 |
| TCF21 | 4.45E-13 | 1.61E-08 | -3.356473905 |
| ITLN2 | 4.95E-13 | 1.79E-08 | -3.651094353 |
| CDO1 | 6.62E-13 | 2.39E-08 | -2.64752933 |
| FMO2 | 1.42E-12 | 5.13E-08 | -3.003420019 |
| RBP2 | 1.45E-12 | 5.24E-08 | -2.845798662 |
| SOX7 | 1.86E-12 | 6.71E-08 | -2.714470691 |
| ADH1A | 4.53E-12 | 1.64E-07 | -3.352295244 |
| MYZAP | 6.73E-12 | 2.43E-07 | -2.873384365 |
| EDNRB | 8.55E-12 | 3.09E-07 | -3.137271794 |
| SDPR | 1.20E-11 | 4.32E-07 | -3.004068363 |
| FABP4 | 1.46E-11 | 5.26E-07 | -3.161134755 |
| NCKAP5 | 1.49E-11 | 5.37E-07 | -2.691220413 |
| SFTPC | 2.04E-11 | 7.36E-07 | -3.399261581 |
| TGFBR3 | 2.17E-11 | 7.84E-07 | -3.135696962 |
| ADH1B | 2.35E-11 | 8.47E-07 | -2.784288856 |
| ACADL | 2.62E-11 | 9.47E-07 | -2.754467034 |
| CAV3 | 2.96E-11 | 1.07E-06 | -2.387357936 |
| CCBE1 | 3.00E-11 | 1.08E-06 | -2.659685162 |
| GKN2 | 3.04E-11 | 1.10E-06 | -2.948443239 |
| TBX4 | 3.80E-11 | 1.37E-06 | -2.298198322 |
| AGTR1 | 3.94E-11 | 1.42E-06 | -2.618403264 |
| WIF1 | 4.29E-11 | 1.55E-06 | -2.830929952 |
| CAV1 | 4.34E-11 | 1.57E-06 | -2.970421002 |
| CPB2 | 4.88E-11 | 1.76E-06 | -2.639610928 |
| AOC3 | 5.94E-11 | 2.14E-06 | -2.951669545 |
| BTNL9 | 6.50E-11 | 2.35E-06 | -2.872303707 |
| SCN7A | 7.26E-11 | 2.62E-06 | -2.562358951 |
| CLEC3B | 8.55E-11 | 3.09E-06 | -2.410681525 |
| C2orf40 | 1.07E-10 | 3.88E-06 | -3.283601032 |
| MAOB | 1.09E-10 | 3.92E-06 | -2.27452536 |
| CSF3 | 1.10E-10 | 3.99E-06 | -2.634120818 |
| FAM150B | 1.14E-10 | 4.10E-06 | -2.712901775 |
| ROBO4 | 1.18E-10 | 4.25E-06 | -2.216368689 |
| RHOJ | 1.34E-10 | 4.85E-06 | -1.976996302 |
| SCGB1A1 | 1.45E-10 | 5.24E-06 | -2.7749403 |
| S1PR1 | 1.68E-10 | 6.06E-06 | -2.335726292 |
| PPARGC1A | 1.69E-10 | 6.12E-06 | -2.077869325 |
| ABCA8 | 1.93E-10 | 6.98E-06 | -2.354045497 |
| ANKRD1 | 1.98E-10 | 7.16E-06 | -3.048297663 |
| MFAP4 | 2.01E-10 | 7.24E-06 | -2.792867036 |
| FIBIN | 2.08E-10 | 7.52E-06 | -2.61215964 |
| ARHGAP6 | 2.34E-10 | 8.46E-06 | -2.038270271 |
| VIPR1 | 2.58E-10 | 9.31E-06 | -2.709066338 |
| FRMD3 | 2.85E-10 | 1.03E-05 | -2.011198079 |
| ANGPT1 | 2.92E-10 | 1.06E-05 | -2.414881939 |
| SLC6A4 | 3.19E-10 | 1.15E-05 | -3.515975257 |
| CAV2 | 4.31E-10 | 1.56E-05 | -2.453482086 |
| AKAP12 | 4.34E-10 | 1.57E-05 | -1.786003996 |
| OGN | 4.86E-10 | 1.76E-05 | -2.297676221 |
| GNG11 | 5.70E-10 | 2.06E-05 | -2.289591733 |
| LPL | 6.06E-10 | 2.19E-05 | -2.437707055 |
| RAMP2 | 6.29E-10 | 2.27E-05 | -2.420463159 |
| LIN7A | 6.54E-10 | 2.36E-05 | -2.0012369 |
| MME | 6.84E-10 | 2.47E-05 | -2.680960499 |
| IGSF10 | 8.22E-10 | 2.97E-05 | -2.41847441 |
| SH2D3C | 8.99E-10 | 3.25E-05 | -1.992919955 |
| ASPA | 1.00E-09 | 3.61E-05 | -2.631036973 |
| RADIL | 1.04E-09 | 3.77E-05 | -2.138055393 |
| SEMA6A | 1.28E-09 | 4.62E-05 | -2.649680581 |
| CLIC5 | 1.58E-09 | 5.70E-05 | -2.179503216 |
| RSPO4 | 1.68E-09 | 6.05E-05 | -2.103595737 |
| C10orf116 | 1.75E-09 | 6.32E-05 | -2.587790386 |
| TNNC1 | 1.82E-09 | 6.58E-05 | -2.647380123 |
| TBX5 | 1.82E-09 | 6.58E-05 | -2.00674378 |
| OLFML1 | 1.83E-09 | 6.62E-05 | -1.767991197 |
| CD93 | 1.87E-09 | 6.75E-05 | -1.886166333 |
| MAMDC2 | 1.95E-09 | 7.06E-05 | -2.33634443 |
| FHL5 | 1.99E-09 | 7.17E-05 | -1.874434016 |
| ACSS3 | 1.99E-09 | 7.19E-05 | -1.794324828 |
| ABI3BP | 2.15E-09 | 7.75E-05 | -2.506557868 |
| CAMK2N1 | 2.26E-09 | 8.15E-05 | -2.546023634 |
| COX7A1 | 2.26E-09 | 8.15E-05 | -1.770744484 |
| CD36 | 2.46E-09 | 8.87E-05 | -2.299206937 |
| CA4 | 2.55E-09 | 9.20E-05 | -3.069578155 |
| ARHGEF26 | 2.57E-09 | 9.27E-05 | -2.015899235 |
| ADRB2 | 2.68E-09 | 9.67E-05 | -1.836963572 |
| KIAA1462 | 2.79E-09 | 0.000100807 | -1.896376419 |
| KANK4 | 2.88E-09 | 0.000103854 | -1.624847331 |
| IL1RL1 | 2.97E-09 | 0.000107293 | -2.239089636 |
| ECSCR | 3.09E-09 | 0.000111481 | -2.051264067 |
| ACVRL1 | 3.14E-09 | 0.000113459 | -2.283542305 |
| CYS1 | 3.25E-09 | 0.000117502 | -1.752766001 |
| NEXN | 3.33E-09 | 0.000120264 | -1.579226215 |
| SOSTDC1 | 3.39E-09 | 0.000122461 | -3.383782464 |
| TBX2 | 3.78E-09 | 0.000136441 | -1.70163574 |
| KLF4 | 3.88E-09 | 0.000139898 | -2.139473056 |
| FLJ30901 | 4.12E-09 | 0.000148713 | -2.112564422 |
| HBB | 4.40E-09 | 0.000158978 | -2.884125259 |
| SLC39A8 | 4.43E-09 | 0.000159988 | -2.338685931 |
| TSPAN7 | 4.61E-09 | 0.000166444 | -2.341264357 |
| PEAR1 | 4.75E-09 | 0.000171439 | -2.16653793 |
| ANXA3 | 5.07E-09 | 0.000182992 | -2.237363719 |
| SPARCL1 | 5.07E-09 | 0.000182992 | -2.472599095 |
| EMP2 | 5.38E-09 | 0.000194384 | -2.022204599 |
| CD5L | 5.82E-09 | 0.000210264 | -2.007440173 |
| FOSB | 5.88E-09 | 0.000212336 | -2.176881422 |
| NOTCH4 | 5.98E-09 | 0.000215878 | -1.790942495 |
| REEP1 | 6.14E-09 | 0.000221617 | -1.654205312 |
| MYOC | 6.66E-09 | 0.000240342 | -2.750144498 |
| PLEKHH2 | 7.16E-09 | 0.000258367 | -2.169461318 |
| DNASE1L3 | 7.62E-09 | 0.000275039 | -2.313605666 |
| ATP1A2 | 8.54E-09 | 0.000308452 | -1.98077275 |
| ANKRD29 | 9.16E-09 | 0.000330535 | -2.404131172 |
| LHFP | 1.03E-08 | 0.000371424 | -2.018158808 |
| DUOX1 | 1.07E-08 | 0.000385928 | -2.269296092 |
| FGFBP2 | 1.08E-08 | 0.000391305 | -2.250408258 |
| PDE8B | 1.09E-08 | 0.000393356 | -1.922058596 |
| TMEM47 | 1.14E-08 | 0.000410504 | -2.15731613 |
| MYCT1 | 1.20E-08 | 0.000431848 | -1.737203143 |
| FEZ1 | 1.21E-08 | 0.000436552 | -1.986568041 |
| VGLL3 | 1.27E-08 | 0.000457179 | -1.495248647 |
| CDH13 | 1.28E-08 | 0.000461438 | -1.969424895 |
| MSRB3 | 1.50E-08 | 0.000541508 | -1.803490835 |
| HEG1 | 1.56E-08 | 0.000563869 | -1.807289215 |
| SVEP1 | 1.61E-08 | 0.000581789 | -1.962393028 |
| ADH1C | 1.64E-08 | 0.000591858 | -2.117076705 |
| SLIT2 | 1.76E-08 | 0.000635255 | -2.070008948 |
| CES1 | 1.76E-08 | 0.000635255 | -1.937254017 |
| KHDRBS2 | 1.80E-08 | 0.00064971 | -1.590118767 |
| GPM6A | 1.86E-08 | 0.000669715 | -2.153182629 |
| COL13A1 | 1.88E-08 | 0.000678667 | -1.853966142 |
| C1orf115 | 1.95E-08 | 0.0007026 | -1.679881518 |
| FHL1 | 1.95E-08 | 0.000705641 | -2.278514979 |
| PIP5K1B | 1.97E-08 | 0.000711755 | -1.941832078 |
| STX11 | 2.00E-08 | 0.000721008 | -1.666551081 |
| LOC400568 | 2.04E-08 | 0.000735643 | -2.707725581 |
| CYYR1 | 2.05E-08 | 0.000739816 | -1.522557336 |
| FZD4 | 2.20E-08 | 0.000795339 | -1.772801349 |
| UACA | 2.29E-08 | 0.000826112 | -1.63931512 |
| RASIP1 | 2.33E-08 | 0.000842948 | -1.960846146 |
| PZP | 2.36E-08 | 0.000850726 | -1.580582344 |
| COL6A6 | 2.39E-08 | 0.000861461 | -2.68936337 |
| MS4A15 | 2.50E-08 | 0.000901792 | -2.453678434 |
| STARD13 | 2.52E-08 | 0.000909291 | -1.539854817 |
| RXFP1 | 2.57E-08 | 0.000928267 | -2.290637042 |
| CLEC1A | 2.60E-08 | 0.00093981 | -1.94517804 |
| SERTM1 | 2.66E-08 | 0.000961883 | -2.446139524 |
| C5AR1 | 2.72E-08 | 0.000983163 | -1.251660069 |
| EPAS1 | 2.92E-08 | 0.001053033 | -2.653348743 |
| IL18R1 | 2.92E-08 | 0.001053435 | -1.623364163 |
| DEFA3 | 3.00E-08 | 0.001081991 | -2.216965119 |
| TEK | 3.06E-08 | 0.001105518 | -2.493636173 |
| TIE1 | 3.08E-08 | 0.001111582 | -1.813691574 |
| SCN4B | 3.12E-08 | 0.001124966 | -2.08150063 |
| HSPB6 | 3.13E-08 | 0.001131666 | -1.932621828 |
| PTN | 3.38E-08 | 0.00122094 | -1.481032722 |
| INMT | 3.42E-08 | 0.001236481 | -1.863191401 |
| AHNAK | 3.48E-08 | 0.001254999 | -1.856616091 |
| PLCL1 | 3.50E-08 | 0.001264875 | -1.262476935 |
| ADAMTS1 | 3.67E-08 | 0.001325497 | -1.857224235 |
| CALCRL | 3.71E-08 | 0.001341025 | -1.983927062 |
| FOXF1 | 3.82E-08 | 0.001377847 | -1.932407115 |
| PTPN21 | 3.90E-08 | 0.001408109 | -2.071852437 |
| SYNPO2 | 4.00E-08 | 0.001444611 | -1.646403229 |
| THBD | 4.35E-08 | 0.00156891 | -1.675230016 |
| PMP22 | 4.56E-08 | 0.001647566 | -1.589905553 |
| SOX5 | 4.81E-08 | 0.00173603 | -1.850687494 |
| PRELP | 4.82E-08 | 0.001739764 | -2.111412529 |
| PEAK1 | 4.83E-08 | 0.001742381 | -1.347823073 |
| LRRK2 | 5.12E-08 | 0.001848485 | -1.705000535 |
| AQP4 | 5.18E-08 | 0.001868966 | -2.149747283 |
| SSTR1 | 5.28E-08 | 0.001905005 | -2.255655116 |
| ID1 | 5.52E-08 | 0.001991895 | -2.039836026 |
| PDE3B | 5.79E-08 | 0.002091919 | -1.378563478 |
| PPARG | 6.07E-08 | 0.002191791 | -1.441003284 |
| TTLL7 | 6.47E-08 | 0.002336609 | -1.862357327 |
| BMPER | 6.47E-08 | 0.002336609 | -1.713074678 |
| HBA2 | 6.56E-08 | 0.002369487 | -2.414677428 |
| LTBP2 | 6.59E-08 | 0.002378221 | -1.425729875 |
| VSIG4 | 6.68E-08 | 0.002411954 | -1.37514109 |
| HHIP | 6.70E-08 | 0.002420198 | -2.486503462 |
| PECAM1 | 6.73E-08 | 0.00242897 | -1.602076747 |
| GPM6B | 6.92E-08 | 0.002497239 | -1.836098794 |
| WFDC1 | 7.13E-08 | 0.002574305 | -1.802024876 |
| FOXF2 | 7.14E-08 | 0.002577683 | -1.61670761 |
| NPR3 | 7.44E-08 | 0.002687162 | -1.713274458 |
| SLCO2A1 | 7.73E-08 | 0.002790895 | -1.708827588 |
| LMO2 | 7.95E-08 | 0.002868415 | -1.689132949 |
| ADARB1 | 8.28E-08 | 0.002988054 | -1.72076024 |
| FBLN5 | 8.33E-08 | 0.003008393 | -1.941442901 |
| RNF144B | 8.56E-08 | 0.003090904 | -1.497721831 |
| PLCB4 | 8.82E-08 | 0.003185974 | -1.445433864 |
| MAL | 8.91E-08 | 0.003214899 | -1.851934987 |
| ITGA8 | 8.91E-08 | 0.003214899 | -2.197297982 |
| TMEM139 | 8.94E-08 | 0.003229003 | -1.66251706 |
| SEMA5A | 9.03E-08 | 0.003261592 | -1.802805622 |
| CCDC68 | 9.43E-08 | 0.00340557 | -1.611882729 |
| DOCK4 | 9.56E-08 | 0.003451482 | -1.476700569 |
| PKNOX2 | 9.64E-08 | 0.003479662 | -2.354379364 |
| KLF2 | 9.98E-08 | 0.003602831 | -1.796549514 |
| NOSTRIN | 1.00E-07 | 0.003614699 | -1.494396274 |
| FAM13C | 1.01E-07 | 0.003630759 | -1.52988838 |
| CLEC14A | 1.01E-07 | 0.003656497 | -1.756050776 |
| FAM162B | 1.03E-07 | 0.003734585 | -1.971164785 |
| SMTNL2 | 1.06E-07 | 0.003809059 | -1.896739465 |
| UGT2B4 | 1.08E-07 | 0.003909457 | -1.691097929 |
| KRT4 | 1.09E-07 | 0.003921976 | -1.777451426 |
| SRPX | 1.12E-07 | 0.004038046 | -1.839601521 |
| SGIP1 | 1.12E-07 | 0.004038046 | -1.316244093 |
| KLF9 | 1.13E-07 | 0.004064182 | -1.900717573 |
| LAMP3 | 1.16E-07 | 0.004202628 | -1.989197315 |
| CYP4B1 | 1.17E-07 | 0.004210464 | -2.016269344 |
| SLC1A1 | 1.20E-07 | 0.004333389 | -1.684468613 |
| PTPRM | 1.25E-07 | 0.004530543 | -1.438360528 |
| ASPRV1 | 1.27E-07 | 0.004573748 | -1.126302252 |
| FAM189A2 | 1.38E-07 | 0.004993817 | -1.830565303 |
| OTUD1 | 1.40E-07 | 0.00504067 | -1.257946671 |
| HLF | 1.41E-07 | 0.005074684 | -1.726370096 |
| HPGD | 1.46E-07 | 0.005264192 | -1.459077927 |
| PRICKLE1 | 1.48E-07 | 0.005329551 | -1.222541464 |
| MEIS2 | 1.50E-07 | 0.0054122 | -1.33169695 |
| PPFIBP1 | 1.60E-07 | 0.005788657 | -1.250657324 |
| SMAD6 | 1.65E-07 | 0.005966848 | -1.743311963 |
| RSPO1 | 1.65E-07 | 0.005971301 | -2.102922109 |
| FLI1 | 1.71E-07 | 0.006186687 | -1.326969727 |
| GIMAP8 | 1.83E-07 | 0.006606469 | -1.753665007 |
| OR5P3 | 1.86E-07 | 0.006706554 | -1.698490938 |
| HBD | 1.88E-07 | 0.006803453 | -2.349008493 |
| RNF182 | 1.92E-07 | 0.006928542 | -2.072592901 |
| ANXA8L2 | 2.02E-07 | 0.007295829 | -2.014182251 |
| PIK3R1 | 2.15E-07 | 0.007764093 | -1.559084998 |
| LRRTM4 | 2.25E-07 | 0.008130656 | -1.67765692 |
| PLAC9 | 2.28E-07 | 0.008248121 | -1.802825073 |
| EMP1 | 2.31E-07 | 0.008343108 | -1.56091347 |
| LIFR | 2.34E-07 | 0.008439004 | -1.575630383 |
| AKAP2 | 2.41E-07 | 0.008691013 | -2.023441253 |
| C7orf58 | 2.44E-07 | 0.008794558 | -1.438359649 |
| SERPING1 | 2.52E-07 | 0.009084995 | -1.207665206 |
| HECW2 | 2.62E-07 | 0.009449563 | -1.37761422 |
| APCDD1 | 2.65E-07 | 0.009555936 | -1.418632668 |
| ST6GALNAC3 | 2.68E-07 | 0.009663305 | -1.549624448 |
| BEX1 | 2.75E-07 | 0.009936134 | -1.586404432 |
| CD52 | 2.78E-07 | 0.010047048 | -1.298925227 |
| COL4A5 | 2.88E-07 | 0.010409928 | -1.575026498 |
| AQP1 | 2.95E-07 | 0.01064642 | -1.145031311 |
| DPYSL2 | 3.11E-07 | 0.011214101 | -1.947582502 |
| DCN | 3.22E-07 | 0.011616869 | -1.702301592 |
| NDRG2 | 3.33E-07 | 0.01203162 | -1.356088256 |
| GIMAP1 | 3.41E-07 | 0.012325924 | -1.467087377 |
| GFOD1 | 3.57E-07 | 0.012876192 | -1.554929424 |
| NECAB1 | 3.57E-07 | 0.012903889 | -2.293477647 |
| MYL9 | 3.69E-07 | 0.013304914 | -1.667353265 |
| EDIL3 | 3.79E-07 | 0.013670819 | -1.385766682 |
| FXYD1 | 3.80E-07 | 0.013725949 | -2.049014537 |
| GJC1 | 3.81E-07 | 0.013743711 | -1.202071183 |
| CAB39L | 3.94E-07 | 0.014220644 | -1.254616372 |
| PTPRB | 4.07E-07 | 0.01471129 | -2.132995563 |
| SNCA | 4.18E-07 | 0.015098238 | -1.765083703 |
| IGFBP6 | 4.21E-07 | 0.015215624 | -1.564295478 |
| DKK3 | 4.32E-07 | 0.015613917 | -1.233893046 |
| GUCY1A2 | 4.33E-07 | 0.015622779 | -1.54935272 |
| SFTPD | 4.35E-07 | 0.01569454 | -1.199421942 |
| RNF125 | 4.40E-07 | 0.015897615 | -1.353527066 |
| KCNT2 | 4.46E-07 | 0.016102875 | -1.472425914 |
| GDF10 | 4.52E-07 | 0.01632896 | -2.392983151 |
| PKIA | 4.66E-07 | 0.016809268 | -1.332829619 |
| KCNA5 | 4.68E-07 | 0.016896691 | -1.600394324 |
| SGCE | 4.68E-07 | 0.016903123 | -1.840794971 |
| SPTBN1 | 4.70E-07 | 0.016984477 | -1.470343318 |
| GIMAP6 | 4.73E-07 | 0.017072627 | -1.499490888 |
| FERMT2 | 4.83E-07 | 0.017428886 | -1.34758354 |
| DACH1 | 4.89E-07 | 0.017647552 | -2.066892812 |
| NR3C2 | 4.91E-07 | 0.017736241 | -1.322748054 |
| CTGF | 4.98E-07 | 0.017974375 | -1.532101087 |
| CDH19 | 5.01E-07 | 0.018104291 | -2.181972882 |
| PTGER4 | 5.13E-07 | 0.018510401 | -1.398292056 |
| BMP2 | 5.27E-07 | 0.019026175 | -2.106222315 |
| SH3D19 | 5.28E-07 | 0.019073651 | -1.225549728 |
| PLCE1 | 5.31E-07 | 0.0191689 | -1.280973943 |
| HSPB2 | 5.32E-07 | 0.019203053 | -1.48117271 |
| SLC46A2 | 5.36E-07 | 0.019360588 | -1.525853738 |
| A2M | 5.62E-07 | 0.020293063 | -2.092281454 |
| C15orf26 | 6.05E-07 | 0.021841447 | -1.032490389 |
| TAL1 | 6.15E-07 | 0.022186118 | -1.680302672 |
| PRR5L | 6.38E-07 | 0.023038452 | -1.253181396 |
| MYLK | 6.49E-07 | 0.023430587 | -1.360573475 |
| NDN | 6.58E-07 | 0.023771115 | -1.450171067 |
| NDNF | 6.79E-07 | 0.024523091 | -1.400453856 |
| CHN1 | 6.91E-07 | 0.024936144 | -1.330599196 |
| IL6 | 7.24E-07 | 0.026148372 | -1.670232307 |
| NR2F2 | 7.28E-07 | 0.026272248 | -1.237549373 |
| SYNM | 7.41E-07 | 0.026769045 | -2.161595692 |
| VEPH1 | 7.47E-07 | 0.026962347 | -1.138422619 |
| PDK4 | 7.59E-07 | 0.02740741 | -1.892917755 |
| AGTPBP1 | 7.63E-07 | 0.027538148 | -1.057395982 |
| C1QTNF7 | 7.70E-07 | 0.027797329 | -1.821441168 |
| GRK5 | 7.86E-07 | 0.028387926 | -1.707177861 |
| NHSL1 | 8.07E-07 | 0.02912416 | -1.759536572 |
| PCDHB15 | 8.09E-07 | 0.029191622 | -1.37005109 |
| PRSS12 | 8.19E-07 | 0.029568034 | -1.61014081 |
| EMCN | 8.28E-07 | 0.029902219 | -2.17090724 |
| BCHE | 8.33E-07 | 0.030083468 | -1.576209397 |
| TUBB6 | 8.37E-07 | 0.030222823 | -1.449191333 |
| HBEGF | 8.69E-07 | 0.031356052 | -1.910490866 |
| NRN1 | 8.81E-07 | 0.031790039 | -1.153657857 |
| MYH11 | 8.84E-07 | 0.031931857 | -1.792940534 |
| CTNNAL1 | 9.32E-07 | 0.033652597 | -1.09522545 |
| KIAA0408 | 9.47E-07 | 0.034176043 | -1.214168099 |
| DENND2A | 9.51E-07 | 0.034344988 | -1.197420604 |
| PLBD1 | 9.66E-07 | 0.034891685 | -1.168979744 |
| SGCB | 9.77E-07 | 0.035286606 | -1.263703474 |
| GALNTL1 | 9.86E-07 | 0.035581797 | -1.282825702 |
| DEFA4 | 9.90E-07 | 0.035740797 | -1.363205376 |
| GATA6 | 9.99E-07 | 0.036060496 | -1.433079735 |
| JAM2 | 1.04E-06 | 0.037484141 | -1.760774629 |
| SFTPA1 | 1.10E-06 | 0.039875203 | -2.085780345 |
| TACC1 | 1.12E-06 | 0.040412541 | -1.548491908 |
| PPAP2B | 1.12E-06 | 0.040590569 | -1.355650326 |
| GABARAPL1 | 1.13E-06 | 0.040769251 | -1.40206118 |
| EBF1 | 1.13E-06 | 0.040858837 | -1.106513376 |
| RBMS3 | 1.13E-06 | 0.040858837 | -1.119219627 |
| C13orf15 | 1.16E-06 | 0.041853816 | -1.713146022 |
| LMCD1 | 1.20E-06 | 0.043340446 | -1.35341105 |
| MARCO | 1.22E-06 | 0.043933782 | -1.84428824 |
| CXorf57 | 1.23E-06 | 0.044386847 | -1.298841895 |
| GLIPR2 | 1.24E-06 | 0.044675854 | -1.21453621 |
| SAMD5 | 1.25E-06 | 0.045016333 | -1.270005254 |
| MGAT3 | 1.25E-06 | 0.045063635 | -2.065085274 |
| STARD9 | 1.26E-06 | 0.045356308 | -1.252854481 |
| SEMA6D | 1.27E-06 | 0.045749001 | -1.472267335 |
| TSHZ3 | 1.29E-06 | 0.046443031 | -1.226155936 |
| OR5P2 | 1.30E-06 | 0.046766113 | -1.515936016 |
| IL20RA | 1.31E-06 | 0.047162221 | -1.267645147 |
| TMTC1 | 1.31E-06 | 0.047348228 | -1.471919108 |
| NTNG1 | 1.32E-06 | 0.047551366 | -1.859510566 |
| ZNF385B | 1.32E-06 | 0.047761396 | -1.253624032 |
| EPB41L2 | 1.37E-06 | 0.049623009 | -1.380660609 |
